# Robust Scan Registration for Navigation in Forest Environment Using Low-Resolution LiDAR Sensors

**DOI:** 10.3390/s23104736

**Published:** 2023-05-14

**Authors:** Himanshu Gupta, Henrik Andreasson, Achim J. Lilienthal, Polina Kurtser

**Affiliations:** 1 Centre for Applied Autonomous Sensor Systems, Örebro University, 702 81 Örebro, Sweden; 2 Perception for Intelligent Systems, Technical University of Munich, 80992 Munich, Germany; 3 Department of Radiation Science, Radiation Physics, Umeå University, 901 87 Umeå, Sweden

**Keywords:** tree segmentation, LiDAR mapping, forest inventory, SLAM, forestry robotics, scan registration

## Abstract

Automated forest machines are becoming important due to human operators’ complex and dangerous working conditions, leading to a labor shortage. This study proposes a new method for robust SLAM and tree mapping using low-resolution LiDAR sensors in forestry conditions. Our method relies on tree detection to perform scan registration and pose correction using only low-resolution LiDAR sensors (16Ch, 32Ch) or narrow field of view Solid State LiDARs without additional sensory modalities like GPS or IMU. We evaluate our approach on three datasets, including two private and one public dataset, and demonstrate improved navigation accuracy, scan registration, tree localization, and tree diameter estimation compared to current approaches in forestry machine automation. Our results show that the proposed method yields robust scan registration using detected trees, outperforming generalized feature-based registration algorithms like Fast Point Feature Histogram, with an above 3 m reduction in RMSE for the 16Chanel LiDAR sensor. For Solid-State LiDAR the algorithm achieves a similar RMSE of 3.7 m. Additionally, our adaptive pre-processing and heuristic approach to tree detection increased the number of detected trees by 13% compared to the current approach of using fixed radius search parameters for pre-processing. Our automated tree trunk diameter estimation method yields a mean absolute error of 4.3 cm (RSME = 6.5 cm) for the local map and complete trajectory maps.

## 1. Introduction

Forestry industries have seen a significant increase in demand for automation in recent decades. The increased demand is due to the harsh and hazardous working environment of forestry machine operators and highly repetitive operations, which necessitates automation solutions. Although the research in automation of local operations involved in tree harvesting has increased [1], the entire chain of operation of forestry machines still needs automation [2]. Furthermore, developing robust navigation and perception algorithms for autonomous forestry machines is challenging due to the exceptional environmental conditions in which they must operate.

For autonomous navigation, Simultaneous Localization and Mapping (SLAM) algorithms are used, which aim to solve a probabilistic problem that involves estimating the posterior probability of a robot’s trajectory and the map of the environment, given a sequence of wheel odometry, perception measurements (scan registration), and global positioning measurements (absolute positioning). SLAM is a well-researched field of robotics [3] and in case of large outdoor environments, current navigation solutions rely heavily on the Global Navigation Satellite System (GNSS) [4]. However, GNSS is unreliable in many forestry robotics applications due to signal blockage and multi-reflection [5]. Therefore, navigation relying on range sensors like sonars, LiDARs, and radars to generate maps and localize them is crucial in such cases [4,5,6].

One of the main components of perception-based SLAM is scan registration, in which robot motion between two different time instances is estimated using visual data. Most state-of-the-art pointcloud registration algorithms use generalized features extracted from the pointclouds and do not employ domain knowledge. However, in forestry conditions, current SLAM algorithms based on these generalized features often fail due to the environmental conditions that impair the accuracy of the acquired pointclouds and the lack of meaningful features to be extracted from the environment. In this paper, we look into improving SLAM by extracting features (tree points) unique to the forest environment and commonly present in the operational conditions of forestry machines. These hand-tailored features possess the strength of minimal assumptions that are reasonable in the application domain and generalize well between different forestry conditions. Hand tailor features also do not require any training data to be collected as one might expect from automated feature extraction-based methods relying on machine learning and deep learning (i.e., [7,8]).

This paper presents a SLAM method explicitly designed for forest environments that utilize low-resolution LiDAR sensors without relying on any other sensors. The proposed method provides additional capabilities including tree diameter estimation and tree mapping. We propose an adaptive method for normal estimation and pointcloud filtration, adjusted to the LiDAR sensor resolution for robust tree detection. The tree point extraction from a single scan of low-resolution LiDAR is challenging due to point sparsity; hence we proposed a heuristic approach to extract trees from filtered pointclouds. The extracted trees are then used to increase the robustness of scan registration. We proposed shape-2-shape registration to get the initial pose estimation and then used the ICP point-2-plane for refinement. Finally, the results of scan registration and detected trees are used for localization and mapping by a landmark-based graph SLAM algorithm implemented in the GTSAM library [9].

The rest of the paper is structured as follows. First, we provide a brief overview of the current state-of-the-art of SLAM algorithms and tree detection algorithms in forestry and orchard environments. Then, Section 3 outlines a detailed description of the proposed method. Section 4 presents experimental results in applying the proposed method to 3 private and open datasets compared to current methods. Finally, in Section 5, we provide additional observations and discuss the limitations of the proposed methods with the paper’s conclusion in Section 6.

## 2. Literature Overview

Outdoor navigation of mobile robots most often relies on the Global Positioning System (GPS) [10]. A GPS signal provides us with the ability to identify the location of a ground vehicle. In ideal conditions, no additional sensory information is required once a GPS signal is combined with an accurately updated map. Unfortunately, these ideal conditions only sometimes occur. To compute its location in three-dimensional space, a GPS receiver must be able to lock onto signals from at least four different satellites [11] for a significant uninterrupted amount of time. Urban environments and heavily foliated areas often contain too many intrusions, blocking the GPS signal and preventing continuous GPS signals from being received [12]. Moreover, even if a proper uninterpreted GPS signal is received, the presence of dynamic obstacles or the unavailability of an up-to-date map requires the mobile ground vehicles to be equipped with complementary sensors such as LiDARs, radars, and sonars to provide local navigation and correction capabilities.

Specifically, in the forest environment, GPS signal absorption in the canopy is reported to lead to large localization errors, generally in the range of several meters [13,14]. Additionally, forest road maps are often less precise than other scenarios, while autonomous vehicles are often required to leave the mapped road to perform operations [13]. As a result, navigation of forestry machines relies most often on the fusion of range sensors [13,15,16,17].

The lack of available maps leads to the simultaneous need to map and localize using range sensors, a process called SLAM (Simultaneous Localization and Mapping). Several state-of-the-art SLAM algorithms have been explored in the forestry domain, including graph-slam [18], fast-slam [19], and semantic-slam [20]. The accuracy of all of those methods relies heavily on one key ability - registration of consecutive scans. Under the assumption that the sensors used for SLAM are range sensors, the scans requiring registration are point clouds. Registration of two pointclouds X and Y means finding the transformation matrix T that aligns the pointclouds. It is an iterative optimization problem in which registration loss is minimized.

Pointcloud registration is typically done in 2 steps - sampling points to find key points and a subsequent feature description. The key points are matched according to the closest point in feature space [21]. When no additional specifications on the environment in which the ground vehicle is to navigate in, key points are often extracted and registered using generic descriptors such as Fast point feature histogram (FPFH) [22], density, curvature, and normal angle features (DCA) [23], and plane-based features (PLADE) [24].

These generalized SLAM methods are also employed for orchard and forestry autonomous navigation. For example, Dong et al., [25] evaluates the performance of the popular ORB-SLAM2 [26] algorithm for navigation and mapping in an apple orchard environment using an RGB-D camera. They show that while some meaningful generalized features can be extracted, and a map can be generated, the accuracy is relatively low and requires correction with the inclusion of domain knowledge such as ground and tree trunk detection.

Given some knowledge about the environment in which the vehicle is to operate in, the feature extraction can be done more accurately. For example, in [7,8]), the authors suggest using deep learning for feature extraction. Given training data collected in similar conditions, these methods provide the registration algorithm with a more meaningful tool; therefore, successful key-point detection is more likely. Alternative methods propose adapting the feature extraction algorithm to the application domain through hand-crafted features or learned descriptors [21]. This is usually done in a somewhat generalized manner, like the intrinsic shape signature method [27] for view-invariant feature extraction and fast pose registration. However, some methods employ the detection of objects for key points extraction, such as the VI-EYE [28] method for pointcloud registration for autonomous cars. The method exploits domain knowledge in traffic scenarios to detect key semantic objects such as roads, lane lines, curbs, and traffic signs to align pointcloud pairs. Similar approaches to employing semantic information for pointcloud registration are attempted in the forestry and orchard domains. For example, the above-mentioned work by Dong et al., [25] employ RGB information collected by the RGB-D camera to detect tree trunks for pointcloud registration correction. Extraction of points belonging to the ground enables the authors to align the pointclouds further. In [29], the authors employed a Lidar for forest inventory mapping relying on tree trunk detection combined with a GPS signal. The system is suggested to employ the GPS signal in open forest areas and LIDAR and IMU-based SLAM for dense areas where a GPS signal is unavailable. They have performed 3D mapping by utilizing a very high-resolution LiDAR sensor. Unfortunately, we did not find additional information about the tree extraction methodology to compare to.

While the hand-crafted feature extraction methods introduce assumptions and limitations, they lift the need for training data. Therefore their generalization between environments is dependent on the assumptions introduced.

In this research, we aim to use domain knowledge about the structure of a forest environment to improve pointcloud registration to increase the performance of autonomous navigation and SLAM in forestry conditions. As described in the methods section, we exploit minimal assumptions about the shape and orientation of trees in relation to the ground plane to extract meaningful key points and align generated pointclouds. Furthermore, we show that the assumptions generalize well between different forest conditions in different datasets.

In our proposed registration and mapping algorithm, segmenting trees in pointcloud data is the key to accurate performance.

The current research on tree detection in visual data acquired in forestry settings focuses primarily on operational monitoring. As a result, most algorithms are developed for fast single-tree detection using the top view from UGVs. For example, in [30], the authors compare 6 methods of tree detection from airborne laser scanning to provide an accurate estimation of biophysical parameters of forests, such as tree height, timber volume, forest structure, and forest distribution. More recent publications (e.g., [31]) aim to employ deep learning to detect and count oil palm trees from remote sensing images.

Terrestrial systems navigating in the forest employ tree detection for forest inventory goals [32] by collecting measures such as tree trunk diameter. In [32], the authors used pointcloud cluster search and point density analysis to perform tree segmentation. They report 97% detection rates and up to 5cm error of diameter estimation. In [33], the authors employ a threshold on the reflectance measures from a LiDAR sensor placed in a single static location, assuming that the trunk surface is Lambertian, of a semi-cylindrical shape. Finally, in [34,35,36], the authors suggest analyzing pointclouds acquired from terrestrial laser scanning by applying a series of pointcloud decomposition and re-joining under the assumptions of cylindrical shape and orientation in relation to the ground, to filter the acquired data. Once the pointcloud is filtered, a tree model is generated, which is then analyzed to extract measures such as stem and branch diameters. Additional similar methods, relying on quantitative structural models, are reviewed by [37] and are deemed the most accurate to date.

Finally, some methods employ additional sensory information registered with the pointcloud to perform segmentation. For example, in [25], the authors employed an RGB-D camera to acquire the pointclouds. The tree trunk segmentation was done on the RGB images that were then matched to the corresponding pointcloud.

## 3. Methods

The key aspect of the proposed method is integrating a well-established basic assumption of forest structure into the generalized SLAM method for navigation in a forest. The method includes detecting trees in a single LiDAR scan and utilizing them as landmarks for pose estimation and the pose-graph SLAM algorithm. Tree stems are chosen for pose estimation due to their saliency, static nature, and robustness across varying weather conditions.

**Figure 1:**
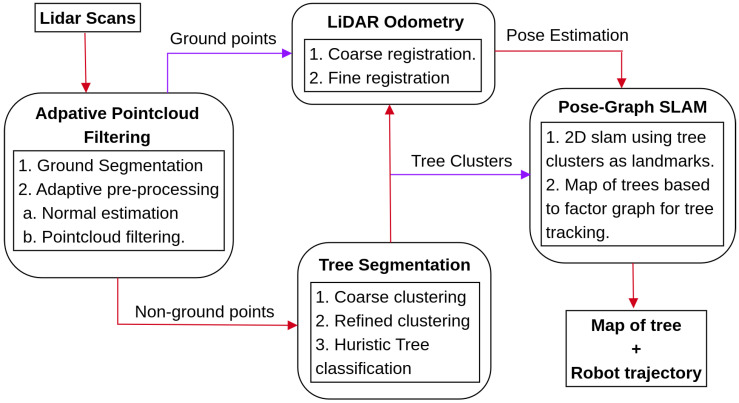
An overview of the system pipeline implemented in this work for creating a map of trees.

Figure 1 illustrates an overview of the proposed approach. The method comprises four major steps, namely: (1) pointcloud pre-processing, (2) tree segmentation, (3) scan registration, and (4) landmark-based pose graph SLAM. The brief description of the proposed approach is that we first filter the pointcloud scan based on point intensity (if available) and segment the filtered pointcloud into the ground and non-ground points. Since the forest environment is complex and the terrain is rough, we further filter the non-ground pointcloud based on point-normal direction and the number of point neighbors using our adaptive filtering approach to remove noisy points. Next, we automatically extract the tree stems from the filtered non-ground pointcloud. We do this by first coarsely clustering the pointcloud using the divide-and-merge method [38], then refining the coarse clusters using Euclidean-based DBSCAN clustering. However, not all refined clusters represent tree stems, so we use a heuristic-based tree classification approach to identify and extract them. Once we have extracted tree stems in consecutive scans, we use them to calculate the initial pose estimate, refined using the ICP point-2-plane approach. Finally, we use the detected tree stems as landmarks and pose estimation from scan registration to create a landmark-based pose graph SLAM for mapping and localization purposes.

Several additional capabilities are developed by employing the proposed method for autonomous navigation. First, a map of the trees is obtained in addition to a navigational map, which can be utilized for applications like tree harvesting, logging, and operational monitoring without additional mapping. Second, the methods can accurately segment trees from low-resolution LIDAR scans, providing additional capabilities like tree trunk size estimation and forest yield evaluation.

A detailed explanation of each step is provided in the next sections, followed by the results section, where we analyze the accuracy of the proposed algorithm for tree detection, tree diameter estimation, and mapping.

### 3.1. Pre-Processing

The pre-processing step focuses on extracting filtered ground and non-ground points, which involves an initial ground segmentation followed by point normal estimation and pointcloud filtering using an adaptive search parameter.

A pseudo-code describing the pre-possessing is available in Algorithm 1. The algorithm first performs intensity-based filtering to remove points belonging to foliage, followed by a ground segmentation using an Approximate Progressive Morphological Filter [39] implemented using PCL’s libraries [40]. The segmented pointcloud has a substantial amount of noise due to the rough terrain of the forest and must be refined further by employing an additional filtering step. The outliers are detected using the point’s normal direction (estimated using adaptive radius search parameters) and the minimum number of neighbors in the point’s vicinity.
**Algorithm 1** Preprocessing—Adaptive Pointcloud Filtering Algorithm **function** Preprocess(*P*)
$P^{\prime }\leftarrow P\left[P_{I}<I_{th}\right]$$P_{G},P_{NG}\leftarrow GroundSeg\left(P^{\prime }\right)$  ▹ Segment ground and non-ground points$XY\leftarrow \left|\right|P_{NG}{\left|\right|}_{XY}$  ▹ Calculate points range in XY-plane$Rings\leftarrow Geomspace(R_{min},R_{max},N_{R})$  ▹ Get inner- outer radius of concentric rings$P_{F}\leftarrow \left\{\right\}$**for** $r_{in},r_{out}\leftarrow Rings$**do**  ▹ Run for ground and non-ground points
$p\leftarrow P_{NG}\left[r_{in}<XY<r_{out}\right]$$\delta_{r}\leftarrow 1.5\times \theta_{V}\times \frac{r_{in}+r_{out}}{2}$  ▹ Calculate search radius$n\leftarrow CalculateNormal(p,\delta_{r})$$p^{\prime },n^{\prime }\leftarrow Filtering\left(\left[p,n\right],\delta_{r},\theta_{th},N_{min}\right)$  ▹ Normal-based and Radius outlier removal Filtering$P_{F}.insert\left(\left[p^{\prime },n^{\prime }\right]\right)$**end for****return** $P_{F}$ **end function**

The algorithm relies on several key parameters which are described as follows:

**Intensity-based threshold ($I_{th}$):** During experiments, it has been found that foliage often produces high-intensity points. Therefore an intensity-based thresholding step is introduced before the initial ground segmentation using a threshold parameter ($I_{th}$) to reject high-intensity points mostly belonging to the foliage. The intensity threshold parameter is different for different LiDAR sensors and has been empirically calculated such that around 10% of the points will be removed and kept constant across the different evaluated datasets.

**Adaptive nearest neighbor search parameter ($\delta_{r}$):** The pointclouds acquired using low-resolution LiDARs in forest environments contain substantial noise due to environmental conditions (i.e., obstructions by foliage) and sensor limitations (low vertical angular resolution). As a result, pre-processing the pointcloud with a fixed-value nearest neighbor search parameters results in less accurate point normal estimation and outlier point filtering. Hence, we proposed an adaptive nearest neighbor search parameter ($\delta_{r}$). The parameter depends on the point distance from the sensor origin and the vertical angle resolution ($\theta_{V}$) of the LiDAR sensor for a more accurate point normal estimation and outlier removal.

Since the estimation of the point normal using adaptive radius search parameter ($\delta_{r}$) for each point is a computationally heavy procedure, we divided the pointcloud into concentric rings in the sensor’s XY-plane and calculated the radius search parameter ($\delta_{r}$) using Equation (1). The radius search parameter ($\delta_{r}$) depends on the inner ($r_{in}$) and outer ($r_{out}$) radius of the ring and the vertical angle resolution ($\theta_{V}$) of the LiDAR sensor.

(1) $$\delta_{r}=1.5\left(\theta_{V}\times \frac{r_{out}+r_{in}}{2}\right)$$

**Angle threshold ($\theta_{th}$):** The pointcloud is filtered by thresholding the point normal using an angle threshold. In the case of ground points the point normal should be vertical (along Z-axis) and for the tree stem points the point normal should be horizontal (XY-plane). The angle threshold is also used in the tree segmentation algorithm as one of the heuristic parameters threshold to classify clusters as trees or not.

**Minimum number of points threshold ($N_{min}$):** The minimum number of points threshold is required for the radius outlier method where a certain point is considered inlier if the number of neighbor points in radius ($\delta_{r}$) is greater than $N_{min}$.

**Figure 2:**
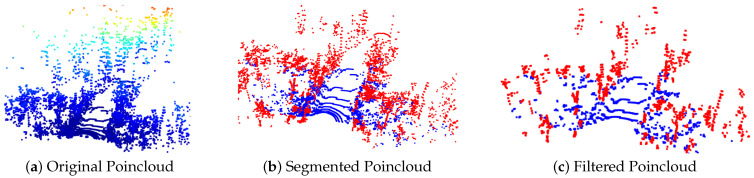
Visualisation of segmented pointcloud (ground and non-ground points using approximate Approximate Progressive Morphological Filter) and pointcloud filtration (point normal based filter and outlier removal filtering) using proposed adaptive search radius approach for a scan from DS2 dataset (Section 4.1). (Ground: blue and non-ground: red).

All threshold parameters mentioned above have been kept constant for pre-processing the pointclouds for all datasets evaluated in this work. After the pre-processing step, a segmented and filtered pointcloud is generated with a point normal. An example is shown in Figure 2) for a 16-channel LiDAR Sensor.

We discuss the impact of using the suggested pre-processing step on tree segmentation from low-resolution LiDARs in the results Section 4.3.

### 3.2. Tree Segmentation

Tree segmentation in low-resolution pointcloud scan serves a dual purpose in this work. First, the segmented trees serve as features to register consecutive pointclouds. Once registration is achieved they are used as static objects/landmarks for map generation and correction in the SLAM phase. Second, accurate tree segmentation allows tree map generation to be utilized in forest monitoring applications where tree trunk and morphological shape extractions are needed.

In this step, the non-ground points obtained from the pre-processing are used for tree segmentation and are processed in three stages. First, non-ground points are clustered coarsely using the divide-and-merge method proposed in [38]. Then the coarse clusters are refined using DBSCAN [41] clustering to obtain the probable tree stem. Finally, the refined clusters are classified as trees or not-tree using a heuristic approach based on four parameters, the number of points in the cluster ($N_{C}$), the minimum height of the cluster (*l*), the angle between the primary axis direction of the cluster, and the sensor’s Z-axis, and the approximation of the height-to-width ratio of the cluster ($\sigma_{hw}$).

**Figure 3:**
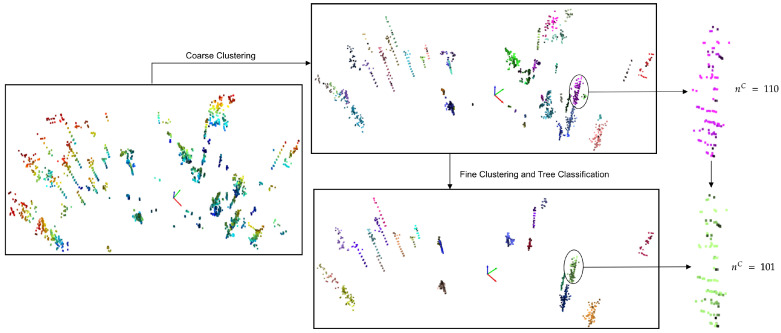
Outputs from the three steps of the proposed tree segmentation pipeline, (i) coarse clustering, (ii) fine clustering, and (iii) tree classification using non-ground points. The non-ground points resulted from the pre-processing of a scan from the DS1 dataset (Section 4.1).

Figure 3 demonstrates the result of coarse clustering, refined clustering, and heuristic-based classification step, and the pseudocode of the proposed tree-stem segmentation algorithm is given in Algorithm 2. Details on each step are as follows.
**Algorithm 2** Tree stem Segmentation**function** SegmentTreestems(*P*)
Clusters←DivideAndMerge(P)  ▹ Coarse clustering of filtered non-ground pointsTrees←{}**for** C in Clusters **do**
$d_{th}^{C}\leftarrow k\times \theta_{V}\times \left|\right|\frac{\sum_{i}^{N_{C}}C_{{XY}_{i}}}{N_{C}}\left|\right|$$FineClusters\leftarrow DBSCAN(C,d_{th})$  ▹ Fine clustering**for** FC in FineClusters **do**
mean,cov←CalculateMeanAndCovariance(FC)U×s×Vh←SVD(cov)$FC_{rot}\leftarrow Vh@FC$  ▹ Orient Pointcloud towards maximum variance$length\leftarrow max\left(FC_{rot}\left[:,0\right]\right)-min\left(FC_{rot}\left[:,0\right]\right)$  ▹ Sub-cluster lengthorientation←arccos(Vh[0,2])  ▹ Orientation of sub-cluster wrt Sensor’s z-axis$\sigma_{hw}\leftarrow s\left[0\right]/s\left[1\right]$  ▹ Estimation of height-to-width ratio**if** $length>l_{th}$ and $orientation<\theta_{th}$ and $\sigma_{hw}<\sigma_{th}$ **then**
Trees.insert(FC)**end if****end for****end for**Trees←MergeCluster(Trees)**return** Trees**end function**

**Coarse clustering**. The non-ground pointcloud is coarsely clustered using a fast and accurate clustering algorithm named *divide-and-merge* [38]. In this method, the pointcloud is first clustered locally (divide step) using a heuristic condition and then merged using a voting scheme that is applied to the edge points of the local clusters. The algorithm was implemented using the code provided by Zhao et al. [38] (https://github.com/placeforyiming/Divide-and-Merge-LiDAR-Panoptic-Cluster) adapted to LiDAR of different resolutions and solid-state LiDAR (accessed on 26 April 2022).

**Refined clustering**. The clusters obtained from the coarse clustering step require further refining to remove noise points or split clusters in two or more probable trees which clustered as one due to proximity during coarse clustering. For this purpose, we used DBSCAN clustering (https://scikit-learn.org/stable/modules/generated/sklearn.cluster.DBSCAN.html (accessed on 15 February 2023)) from Scikit-Learn python library [42] as it fits the requirement in which additional noise points can be removed and clustering can be performed based on Euclidean distance. For refinement, euclidean-based clustering is sufficient as we calculate an adaptive distance threshold for each cluster separately based on cluster distance from the sensor origin in XY-plane and the vertical angle resolution $\theta_{v}$ of the LiDAR as given in Equation (2). The cluster distance in the equation is the mean of cluster points ($p_{i}$) in the cluster in XY-plane. As the distance between points in the cluster is not even, we increase the distance threshold by a factor of *k*, and based on experiments we found that k=1.25 worked best for different lidar sensors. For a core point consideration or noise point removal, the minimum number of neighbor thresholds was two in all experiments. The minimum number of neighbors threshold is kept lowest possible (=2) due to the low number of points in the clusters for low-resolution LiDARs. Using a larger value could potentially remove core points.

(2) $$d_{th}^{C}=k\times \theta_{V}\times \left|\right|\frac{1}{n^{C}}\sum_{i}^{n^{C}}p_{i}\left|\right|$$

**Tree classification**. The refined clusters do not guarantee to be tree stems hence classified as trees or not-trees using a set of heuristic criteria based on: the minimum number of points in the cluster, the axis of maximum variance in the cluster being along the sensor’s Z-direction, the length of the cluster along the axis of maximum variance, and the approximation of the height-to-width ratio of the cluster. These parameters are estimated by doing a singular value decomposition of the cluster covariance. Finally, the tree stem clusters were merged based on their proximity in the XY-plane to group tree clusters from the same tree.

### 3.3. Local Pointcloud Registration—LiDAR Odometry

Once the ground points and segmented tree stems are available from the previous steps, they are utilized for consecutive scan registration for LiDAR odometry.

The LiDAR odometry is a collection of sensor poses as a function of time estimated by integrating the pairwise scan registration of consecutive scans. The proposed registration procedure consists of two primary steps: (1) estimating the initial pose guess and (2) refining 3D pointcloud registration.

**Initial Pose Estimation:** Tree correspondences between the previously detected tree clusters in the target scan (T) and the source scan(S) are used to estimate the initial pose in the XY-plane and rotation along the Z-axis. The 1-to-1 tree correspondence between the detected trees is made using a mutually exclusive nearest-neighbor approach based on the Euclidean distance of the tree clusters in XY-plane. The initial pose guess ($\Theta_{init}$) is then calculated by minimizing the shape-2-shape cost function given in Equation (3), where the shape of the clusters is approximated as a Gaussian distribution (mean μ and covariance Σ) of the cluster points. The minimize function of the SciPy library (https://scipy.org/ (accessed on 15 February 2023)) is used for optimization.

(3) $$f_{s2s}=-\sum_{i}\exp-\frac{1}{2}\;\mu_{i}^{T}\;\Sigma_{i}^{-1}\;\mu_{i}$$

Here, $\mu_{i}=R\;\mu_{S_{i}}+t-\;\mu_{T_{i}}$, and $\Sigma_{i}=R\;\Sigma_{S_{i}}R^{T}+\;\Sigma_{T_{i}}$.

**Refined registration**. Many state-of-the-art registration methods could be used to refine registration once an initial pose estimate is made. In our experiments, we chose to employ the very common Iterative Closest Point (ICP) point-2-plane method [43] due to the availability of point normal and ICP’s capability of robust registration for good pointcloud overlap and easy initial pose. Other methods of refined pointcloud registration can be employed at this step like ICP point-to-point, NDT point-to-distribution [44], and others. The algorithm performs registration on the pointcloud obtained by merging the segmented tree clusters and ground points, with the use of the initial pose estimate.

### 3.4. Pose-Graph SLAM

**Figure 4:**
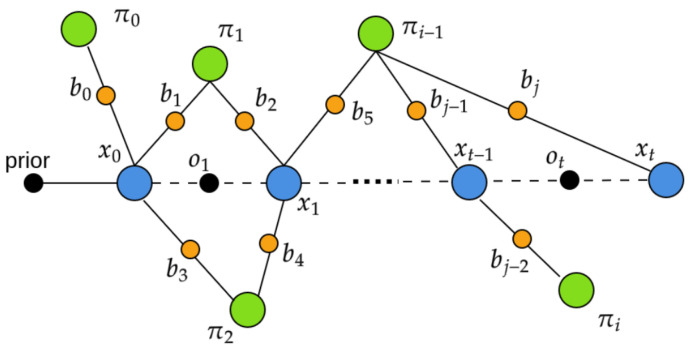
Pose graph structure for SLAM. Each node $x_{t}$ represents the pose of the sensor at time t, with constraints being relative pose transformation $o_{t}$ and landmark bearing $b_{j}$ for the trees $\pi_{i}$.

LiDAR odometry provides accurate sensor trajectory estimation for short distances, but drift accumulation leads to incorrect tree associations in maps for longer distances. To address this, we propose a landmark-based pose-graph SLAM approach that uses tree cluster positions $\pi_{i}$ and relative transformations $o_{t}$ between poses as constraints in a factor graph. Sensor poses represent the nodes $x_{t}$ of the graph, and tree positions are added as range and bearing factors $b_{j}$. Figure 4 represents the landmark-based graph SLAM approach. Our implementation uses the GTSAM library [9] for creating factor graphs and is optimized using the iSAM2 approach [45] (part of GTSAM library).

**2D Pose Graph SLAM:** In this work, we have used landmark-based 2D pose graph SLAM where segmented trees are considered landmarks. The range and bearing factor for the landmark are calculated using the mean of the tree cluster points. The tree stems position is consistent in the XY-plane hence the 2D SLAM with detected trees is evaluated. The pose graph is associated with a tree clusters map that tracks the number of tree clusters and their position on the map. New tree clusters were added if no association could be found in the existing map, and associated tree clusters were used to update the existing tree clusters. However, this approach is not suitable for landmark-based 3D pose graph SLAM as the height of the tree clusters from the ground is highly varied between scans.

**3D Mapping:** For 3D mapping, we did SLAM where two maps are maintained. The first map is a list of detected trees in the map frame and the second map is a collection of ground points. The map of trees is used for coarse registration by matching the trees detected in the current frame to the trees available in the map. For refined registration, ICP point-2-plane registration is done for the matched tree points and ground points. After registering the current scan to the map, the tree map is updated with matched and new trees in the current scan. The result of 3D mapping from our approach is a tree map and the ground map which can be used for DBH estimation.

## 4. Results

### 4.1. Datasets

Three different datasets (DS1-DS3) of short and long sequences collected using low-resolution LiDARs in different environments are used to evaluate the proposed approach. The evaluation includes checking the effect of the adaptive preprocessing on tree detection and scan registration for navigation in a tree rich/forest environment. The datasets used are given below, with more details on the properties of LiDAR sensors presented in Table 1.

- DS1: We have collected a short sequence of the dataset in a natural forest with rough terrain using a 16-channel Velodyne lidar (Model: VLP-16 puck LiDAR) mounted on a harvester machine at a height of around 1 m from the ground level.
- DS2: The second dataset was collected using a solid-state lidar (SSL, Model: RS-Lidar-M1) sensor mounted on a Husky robot in a forest park with relatively flat ground. The sensor was mounted at a height of around 0.45 m from the ground.
- DS3: The third dataset used for evaluation is publicly available, Montmorency dataset [46], which contains long sequences in a different forest environment with a 32ch-lidar sensor mounted on a husky platform. The dataset was collected in four forest sites and has the ground truth for tree positions and diameter. We evaluated our proposed approach on the dataset of forest site id 1 belonging to the Young forest with two trajectories.

### 4.2. Evaluation Measures

The evaluation of the proposed method using the datasets mentioned above aims to measure the accuracy of sensor trajectory generated using LiDAR odometry, the accuracy of landmark-based SLAM, tree detection, and diameter estimation from single versus accumulated scans.

**Tree detection and size estimation**. To compare the effectiveness of the proposed adaptive search radius parameter versus the fixed search radius parameter, we report the average number of coarse clusters and tree stems detected in single scans across three datasets.

We evaluated our approach for tree diameter estimation using single and accumulated scans from the DS3 dataset, which includes ground truth information for tree diameter at breath height (DBH) and tree position in the map. We first detected tree stems using our adaptive search parameter approach, then performed cylinder segmentation using the PCL Library’s SACSegmentation algorithm and estimated the tree diameter using a cylinder-fitting method (https://github.com/xingjiepan/cylinder_fitting (accessed on 15 February 2023)). We randomly sampled 50 scans for tree extraction and diameter estimation. For single scans, we reported the diameter estimated from the cylinder fitting. In contrast, for accumulated scans, we reported the diameter estimation from the cylinder fitting and the DBH estimation using points at the height of 1.15–1.45 m from the ground. The latter is beneficial for tree harvesting machines that require on-the-go diameter estimation.

**Effect of feature-based registration on LiDAR odometry** Since none of the datasets has ground truth for robot/machine navigation, we compared the results of our approach’s odometry and landmark-based SLAM with those of state-of-the-art SLAM methods such as LIO-SAM (for DS1 and DS2) or the trajectory provided with the dataset (DS3). The trajectory generated using our approach depends only on the low-resolution LiDAR sensor. In contrast, the LIO-SAM algorithm and the trajectory provided with the DS3 dataset rely on sensor fusion algorithms. We report RMSE between the trajectories generated using our approach and those provided by the LIO-SAM algorithm or the dataset.

For the DS3 dataset, we also report the overlap between the estimated and ground truth tree positions and the error in DBH estimation for the entire map.

### 4.3. Effect of Adaptive Filtering on Tree Detection

**Figure 5:**
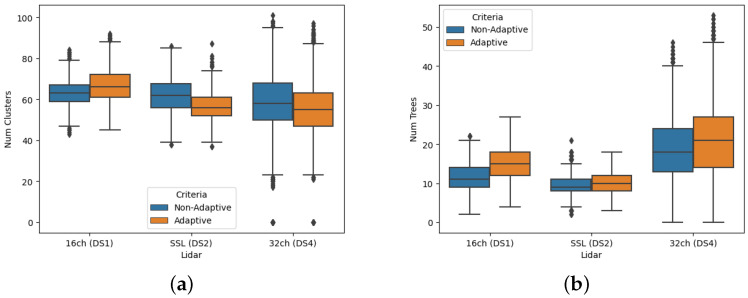
Box plots showing the effect of the adaptive approach on (**a**) the number of clusters detected and (**b**) the tree detection for different Lidar sensors. The number of detected trees is higher for the adaptive approach, which results from more accurate normal estimation and pointcloud filtering.

This section presents the impact of the proposed adaptive normal estimation and pointcloud filtering approach on tree detection using low-resolution LiDARs. The adaptive approach adjusts the search radius for normal estimation and outlier removal based on the LiDAR sensor resolution and the point distance from the sensor, resulting in improved pointcloud filtration, clustering, and tree segmentation. Figure 5 shows the boxplot of the number of coarse clusters and segmented tree stumps detected using the adaptive and non-adaptive approaches for DS1, DS2, and DS3 datasets. The results indicate that the number of coarse clusters detected is higher for the non-adaptive approach. However, the number of detected trees is higher for the adaptive approach, which results from more accurate normal estimation and pointcloud filtering.

**Figure 6:**
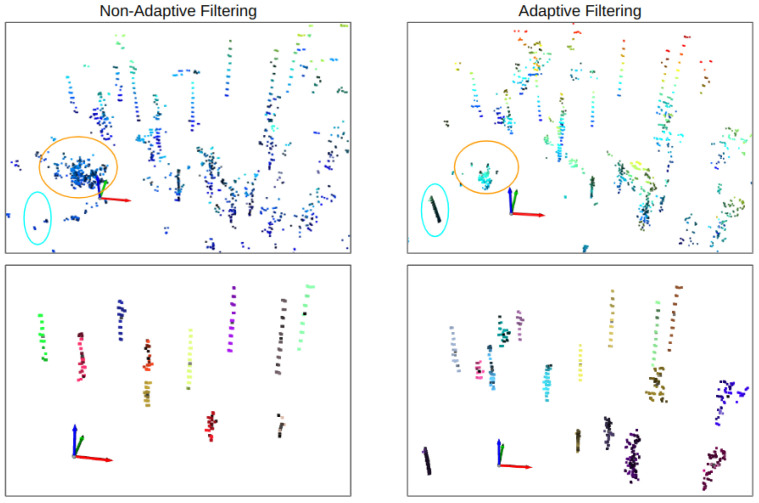
Visualization of the effect of adaptive and non-adaptive filtering approaches on tree segmentation for a single pointcloud scan of 16-channel LiDAR scan in natural forest. The noise for non-adaptive filtering is prominent near the sensor compared to the area far from the sensor—this noise results in incorrect clustering and fewer detected trees as shown in the bottom row.

Figure 6 illustrates the effect of the adaptive and non-adaptive filtering approaches on tree detection. The figure shows that the noise after the non-adaptive filtering approach is more prominent, especially near the sensor area (circled in orange), which leads to fewer detected trees due to incorrect point normal estimation and filtering which also results in the removal of core points (circled in blue). On the other hand, the noise in pointcloud, filtered using our adaptive approach, is less for the far and near areas around the sensor due to the adaptive search radius. This adaptive approach helps with better clustering and hence better tree segmentation as evident from Figure 5. However, some outlier points may still exist in the refined clusters, and the adaptive approach can sometimes produce discontinuous tree stem points, resulting in multiple clusters for a single tree.

### 4.4. Tree Diameter Estimation from Single Scan vs. Accumulated Scans

In this section, we presented results for tree diameter estimation using the DS3 dataset and compared the diameter estimation for the detected trees using a single scan and accumulated scans. The scans are accumulated using LiDAR odometry results for 25 scans (2.5 s). As the LiDAR’s field of view covers the ground in the DS3 and the robot is moving continuously at a speed of <1 m/s, the robot moves roughly 2 m (based on the experiment with LiDAR odometry) during this time period enough ground points are accumulated around segmented trees. With the assumption of the robot moving continuously at all times, we decided to use 2.5 s for scan accumulation in our experiments, however, a larger time period could also be used.

To determine the correspondence between the detected trees and the ground truth, we first orient the detected trees with the reference map provided with the DS3 dataset. Then, using a distance threshold of 10 cm, we could find the correspondence between detected trees and the provided ground truth position in the XY-plane of the reference map. As a result, we found 109 and 236 correspondences for trees extracted from the single and accumulated scans, respectively, with a mean absolute error (MAE) of approximately 6.3 cm and 5.9 cm between the estimated tree diameter and ground truth DBH. However, the MAE of the DHB for the accumulated scan is 4.4 cm.

**Figure 7:**
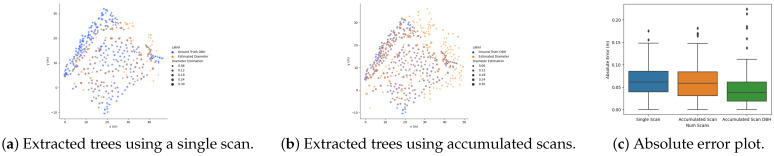
Error plot for tree stem diameter estimation vs. the number of scans used for tree stem extraction.

Figure 7 shows the absolute error boxplot between the estimated tree diameters and ground truth DBH. From the plot, we can see that the absolute error for tree diameter estimation is less for combined scans than tree diameter estimation from a single scan due to more points for the detected trees, which results in more accurate tree position and diameter estimation compared to ground truth.

### 4.5. LiDAR Odometry and Pose-Graph SLAM

This section presents the result of our feature-based registration approach using low-resolution LiDARs in forest environments. As no ground truth for robot/machine trajectory is available, we have compared the results of our approach with other SLAM methods and the error in estimated distance traveled. For the DS1 and DS2 datasets, we compared our results with the trajectory generated using the LIO-SAM SLAM algorithm. In the case of the DS3 dataset, we used the odometry available in the dataset. We have reported the number of tree correspondences found and the DBH error for the DS3 dataset as an additional measure.

**Figure 8:**
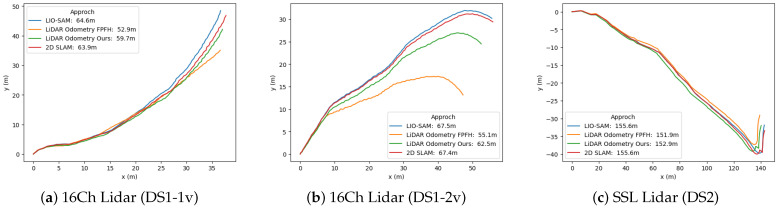
Comparison of the LiDAR odometry and 2D Pose graph SLAM using the proposed approach and results from state-of-art SLAM approach.

**Tree-feature-based Registration vs. FPFH-based Registration:** The estimated trajectories obtained from the proposed tree-feature-based registration (Green) and FPFH-feature-based registration (Orange) are shown in Figure 8. Both methods perform coarse and refined registration, with the only difference being the features used for coarse registration. FPFH-feature registration used voxel sizes of 0.25 m and 0.15 m for DS1 and DS2 datasets, respectively.

Table 2 reports the RMSE error for the LiDAR odometry generated using FPFH-based registration and tree-feature-based registration, along with the tree-based pose graph SLAM approach, which is compared using the trajectory generated by the LIO-SAM approach. We can observe that the tree-feature-based registration outperforms FPFH-feature-based registration for low-resolution LiDAR sensors (16ch LiDAR), with a decrease in RMSE by over 3 m (from 5.9 m to 2.6 m for DS1-v1 and from 8.9 m to 3.3 m for DS1-v2). However, for the solid-state LiDAR sensor, the RMSE from both methods is almost similar. The trajectories from these methods are also shown in Figure 8.

**Lidar odometry and 2D pose graph SLAM:** The trajectory generated by LiDAR Odometry and 2D pose graph SLAM using tree position as a landmark for DS1 and DS2 dataset is shown in Figure 8. The performance of the proposed method is compared with the state-of-art SLAM method, and Table 2 shows that the 2D pose graph SLAM can effectively correct the drift from LiDAR odometry. The RSME is reduced, and the estimation of the distance traveled is improved using the proposed method.

**3D Mapping with low-resolution LiDAR:** 3D Mapping of the forest is done by using only LiDAR sensor data. Two different maps are maintained, the first map is a list of detected trees in the map frame and the other map is made of ground points and mapping as described in Section 3.4.

**Figure 9:**
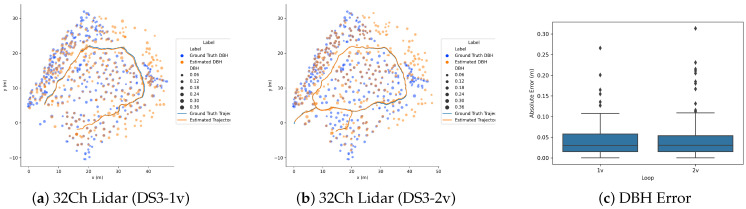
Result of 3D mapping using LiDAR sensor with the proposed approach for DS3 dataset. (**a**,**b**) shows the estimated trajectory along with the position of the trees in the XY-plane and (**c**) shows the corresponding DBH error plot.

Figure 9 show the position of detected trees and the estimated DBH for the complete trajectory. The number of tree correspondences found for a DS3-1v is only 90, much lower than those found for DS3-2v 156. The MAE of DBH estimation for the tree correspondences is 4.4 cm (RMSE = 6.3 cm) and 4.3 cm (RMSE = 6.5 cm) for DS3-1v and DS3-2v, respectively, and the corresponding absolute DBH error plot is given in Figure 9c.

## 5. Discussion

Analysis of the results presented above shows a considerable reduction in RMSE of the LiDAR odometry for the low-resolution LiDAR sensor (VLP-16) using the proposed approach. The RMSE is over 3m less than the state-of-art FPFH feature-based registration method while the RMSE of LiDAR odometry for SSL LiDAR is approximately 3.7 m for both approaches. We obtained a mean absolute error(MAE) of 4.3 cm (RMSE = 6.5 cm) for DBH estimation on the DS3-v2 dataset (most complex forest site in Montmorency dataset [46]) for the fully automated system while the best RMSE for DBH estimation reported in [46] was 3.45 cm for their whole dataset. The comparison of DBH estimation is only partially accurate as in [46] the trees were extracted manually, the DBH estimation reported is for the whole dataset, and the DBH estimation method is different compared to the one employed in our work. However, there is a considerable margin for improvement in the DBH estimation.

Nevertheless, the proposed method possesses several limitations worth mentioning and discussing. First, the method relies on the basic assumption of the presence of trees in the scene. The trees are assumed to be of cylindrical tree trunk shape growing somewhat perpendicular to the ground. These assumptions generalize well in many acquisition forestry environments, as presented in the analyses of the three datasets. The method can not be employed in areas with no trees but can handle a few scans without trees while mapping.

The basic assumption of growing orientation comes into realization in calculating the angle between the tree and the ground to classify the trees. This step relies on the accurate extraction of the ground plane. Segmenting the ground from the pointcloud is also necessary for accurate tree diameter at breath height (DBH) estimation. The ability to extract the ground depends on the orientation of the LiDAR with the ground. This assumption can sometimes fail because the forest terrain can be rough. We address the problem by registering multiple consecutive frames for the LiDAR sensor’ FOV to cover some ground to get a reasonable amount of points to estimate the ground plane orientation near each tree.

Additionally, our method uses analytical methods to extract trees from a single scan to make our method feasible with most LiDAR sensors which is a time-consuming part of the algorithm. In addition, the algorithm was implemented in Python and is not fully optimized for real-time control applications. However, roughly 1.5 scan/second processing speed for the whole SLAM pipeline was achieved. In addition, the proposed analytical tree detection approach can be replaced with learning-based methods, explicitly deep-learning architectures, at the expense of losing generality and improving the processing time. Nevertheless, one can assume such models will be available, and integrating them into the scan registration network should significantly increase accuracy and speed.

Given the rough terrain examined in the datasets, we show that the method fails to solve the problem of 3D slam when relying exclusively on LiDAR sensors. Furthermore, pose-graph 3D SLAM using the tree position as a landmark did not work well on the evaluated dataset due to the significant deviation of the tree key point along the Z-axis (height of key points from the ground) in scans. Therefore, for 3D trajectory extraction, the need to integrate IMU information is apparent.

Finally, we observe that the proposed method is robust to a tilted sensor (up to 25° between the sensor’s z-axis and gravity direction) for accurate tree detection and mapping from a single scan frame. However, orienting the pointcloud along the gravity (using IMU and magnetometer sensor data) results in better tree stem extraction. We also observe that while a single acquisition of the area is sufficient for navigation purposes, repeating acquisition along the same paths produced a better estimation of tree position and tree trunk estimation, leading to a better tree map. This observation was also made previously by [46].

## 6. Conclusions

This paper presents a method capable of increasing navigation and tree size estimation accuracy for the automation of forestry machines compared to the current state-of-the-art. Results show that the method increases registration accuracy between consecutive scans by 3–5 m in RMSE compared to odometry using state-of-the-art FPFH. The increased accuracy benefits autonomous navigation and forest inventory monitoring through tree detection and size estimation. Therefore, including the basic assumption of forest morphology benefits registration and generalizes well between data sets. The idea of integrating trees as special features to be registered has been shown to generalize well and provide the ability to perform accurate navigation using low-resolution LiDARs. Developments in high-resolution LiDAR availability and tree detection algorithms may further increase the algorithm’s ability to generate accurate 3D localization maps.

## Figures and Tables

**Table 1:** Properties of different LiDAR sensors and the datasets used in this work.

	Ours		Montmorency (Forest Site: 1)	
Dataset:Lidar Type	DS1:16ch	DS2:SSL	DS3:32ch	
$\theta_{H}$ (°)	0.2	0.2	0.2	
$\theta_{V}$ (°)	1.875	0.4	1.25	
FOV up (°)	15.0	12.5	10.0	
FOV down (°)	−15.0	−12.5	−30.0	
Range Projection Res	16 × 1800	127 × 620	32 × 1800	
$I_{th}$	NA	60	180	
Num of Scans	669	710	3457	4027
Travel Time (s)	67	71	345	402

**Table 2:** RMSE for the LiDAR odometry using FPFH and Tree-based features, and the proposed tree-based correction of 2D SLAM. The trajectory generated using the LIO-SAM approach is used as a ground truth for RMSE calculation.

	RMSE (m)		
**Dataset**\**Methods**	**FPFH-LiDAR Odometry**	**Ours-Lidar Odometry**	**Ours-2D SLAM**
DS1-1v -16 Ch	5.9	2.6	0.7
DS1-2v -16 Ch	8.9	3.3	1.5
DS2 - Solid state	3.7	3.8	2.8

## Data Availability

DS1: Data available on request due to restrictions. DS2: Data available on request due to restrictions. DS3: 3rd Party Data Available at https://norlab.ulaval.ca/research/montmorencydataset/.
